# Physical training minimizes immunological dysfunction, oxidative stress and tissue destruction on experimental periodontitis in rats

**DOI:** 10.1371/journal.pone.0303374

**Published:** 2024-06-06

**Authors:** Railson de Oliveira Ferreira, Vinicius Ruan Neves dos Santos, José Mário Matos Sousa, Beatriz Rodrigues Risuenho Peinado, Deiweson Souza-Monteiro, Leonardo Oliveira Bittencourt, Maria Laura de Sousa Lima, Cassiano Kuchenbecker Rösing, Fabrício Mezzomo Collares, Aurigena Antunes de Araújo, Rafael Rodrigues Lima

**Affiliations:** 1 Laboratory of Functional and Structural Biology, Institute of Biological Sciences, Federal University of Pará, Belém, Pará, Brazil; 2 Postgraduate program in Oral Science / Department of Biophysics and Pharmacology, Centro de Biociências, Federal University of Rio Grande do Norte, Natal, Rio Grande do Norte, Brazil; 3 Faculty of Dentistry, Department of Periodontology, Federal University of Rio Grande do Sul, Porto Alegre, Rio Grande do Sul, Brazil; 4 Department of Conservative Dentistry, Dental Materials Laboratory, School of Dentistry, Federal University of Rio Grande do Sul, Porto Alegre, Rio Grande do Sul, Brazil; International Medical University, MALAYSIA

## Abstract

The objective of this study is to investigate the effects of a moderate intensity physical training protocol, on alveolar bone morphology of rats submitted to ligature-induced periodontitis. Twenty-eight male Wistar rats were divided into four groups, considering the presence/absence of periodontitis and presence/absence of training. The training protocol was performed on a treadmill, 30 min/day, 5 days a week, for 4 weeks. In the experimental periodontal breakdown, with/without training, ligatures were placed on the lower first molars on the 14th day of the experiment, and were followed until the end of the protocol. At the end of the experiment, animals were euthanized and samples of plasma and mandibles were collected for immunoenzymatic evaluation of interleukins (IL)-1β, IL-6, TNF-α and IL-10, evaluation of serum concentrations of C-reactive protein, analysis of lipid peroxidation (LPO) and reduced glutathione, histological and microtomographic analyses were performed. Physical training resulted in a reduced levels of IL-1β, IL-6, TNF-α C-reactive protein and LPO and an increase in the levels of IL-10 in rats with periodontitis (p<0.05); a reduction in the inflammatory infiltrate and decreased fiber degradation was identified in histological analysis. Additionally, it was shown a decrease in vertical bone loss and an increase in the bone volume/trabecular volume ratio was identified in periodontitis+physical training group (p<0.05). Based on the results, the practice of frequent physical exercise, at moderate intensity, can contribute to the reduction of damage related to the disproportionate inflammatory response in periodontitis.

## Introduction

Engagement in the recommended physical activity or exercise level is important to a healthy lifestyle. It exerts beneficial effects in reducing cardiovascular risks, negative metabolic conditions, systemic inflammation, and mortality rates [1]. The American College of Sports Medicine supports a dose-response relationship between physical activity and health outcomes and with 150min per week of moderate-intensity exercise is associated with lower rates of cardiovascular disease and premature mortality, especially in previously non-active individuals [2]. This position plays a pivotal role in guiding evidence-based practices regarding health initiatives related to physical activity, and exercise prescription. Also, it provides a framework for interdisciplinary collaboration and translational research.

Defined as any body movement that produces an increase in energy expenditure by skeletal muscles physical activity encompasses exercise, which is a planned and structured activity aiming to enhance physical fitness through methodological variables of training such as volume, intensity, frequency, and type [3]. Consistent practice of both concepts reportedly balances systemic immune host-response and also aids in controlling oxidative stress radicals by enhancing antioxidant levels [4,5].

Regarding immune system effects, interleukin-6 (IL-6), a pro-inflammatory cytokine also known as a myokine, interplays a role in both innate and adaptative immune responses activating macrophages and promoting lymphocyte B differentiation [5]. In a recent systematic review of 18 trials, moderate-intensity exercise has been shown to induce acute IL-6 synthesis from 1.29 to 4.20 times, primarily from skeletal muscle fibers [5]. However, the pro-inflammatory triggered by the IL-6 response is balanced by an upregulation of an anti-inflammatory cytokine, interleukin-10 (IL-10) [6]. Training protocols of moderate intensity ranging from 8 to 12 weeks have been associated with post-exercise elevated levels of IL-10 may contribute to a reduction of pro-inflammatory cytokines (interleukin-1b and TNF-a), the resolution of inflammation, tissue repair, and adaptation to exercise stress in patients with inflammatory conditions such as lupus erythematosus [7], stroke [8], and Bowel’s syndrome [9].

Periodontal disease stands as the sixth most prevalent inflammatory disease in humans which presents clinical signs of inflammation in tooth-supporting tissues comprising the gingiva, periodontal ligament, and alveolar bone [10]. Its pathogenesis is multifactorial, characterized by a significant inflammatory profile resulting from interactions among bacterial pathogens, host response, and individual health habits [11]. In the case of gingivitis, signs and symptoms, such as bleeding upon stimulation are restricted to the gingiva (free gingiva, interdental gingiva, attached gingiva) and do not extend beyond mucogingival junction (upper limit of gingiva and lower limit of alveolar mucosa). Halitosis may be present. However, when the inflammatory signs manifest in deeper tissues (cementum, periodontal ligament, and alveolar bone), periodontitis ensues, marked by symptoms including bleeding, gingival recession, tooth mobility, destruction of the periodontal ligament, alveolar bone resorption and, at advanced situations, tooth loss may occur [12]. Periodontal treatment, oral hygiene, and self-care measures are imperative to maintain good periodontal health [11].

Recently, researchers have identified exercise as a potential strategy for controlling risk factors associated with periodontal disease [13]. Although the precise mechanism underlying the interaction between exercise and periodontitis remains unclear, some reports emphasize glycemic control and the adaptation of host immune response [14–16]. Translational research reported a reduction of gene expression of pro-inflammatory cytokines and an improvement of glycaemic status in rodents submitted to aerobic training protocols [14,15]. These findings were clinically supported by a blinded randomized controlled trial involving 47 participants, which evaluated the effects of a 26-week protocol of aerobic, resistance, and combined aerobic-resistance training on clinical assessment levels of glycosylated hemoglobin and high-sensitivity C reactive protein [16]. The intervention group showed reductions in bleeding on probing and severity of periodontitis, as well as HbA1c levels, while hsCRP levels significantly increased in the control group [16].

Moreover, physical activity has been implicated in stimulating biochemical responses that lead to a reduction in oxidative stress. Research has shown that regular physical activity is related to increased levels of antioxidant enzymes in saliva, gingival crevicular fluid, and blood, including uric acid, reduced glutathione (GSH), and catalase [4]. These antioxidant parameters play a crucial role in maintaining oxidative biochemistry balance during exercise and inflammatory processes such as periodontal disease. Specifically, GSH plays a significant role in downregulating NF-κB transcription factors, thereby inhibiting osteoclast activity and differentiation [14]. Therefore, physical activity may contribute to a decrease in the prevalence of periodontal disease by influencing the host immune response [13].

Based on these previous findings, this study seeks to investigate the impact of a moderate-intensity physical training protocol on alveolar bone morphology and inflammatory markers in rats submitted to ligature-induced periodontitis.

## Methods

### Ethical procedures

The experimental design and procedures followed the Guide for the Use and Care of Laboratory Animals (ARRIVE guidelines for reporting in vivo experiments). The Ethics Committee for Animal Use of the Federal University of Pará approved this study (protocol# CEUA-UFPA Nª3159181219).

**Fig 1:**
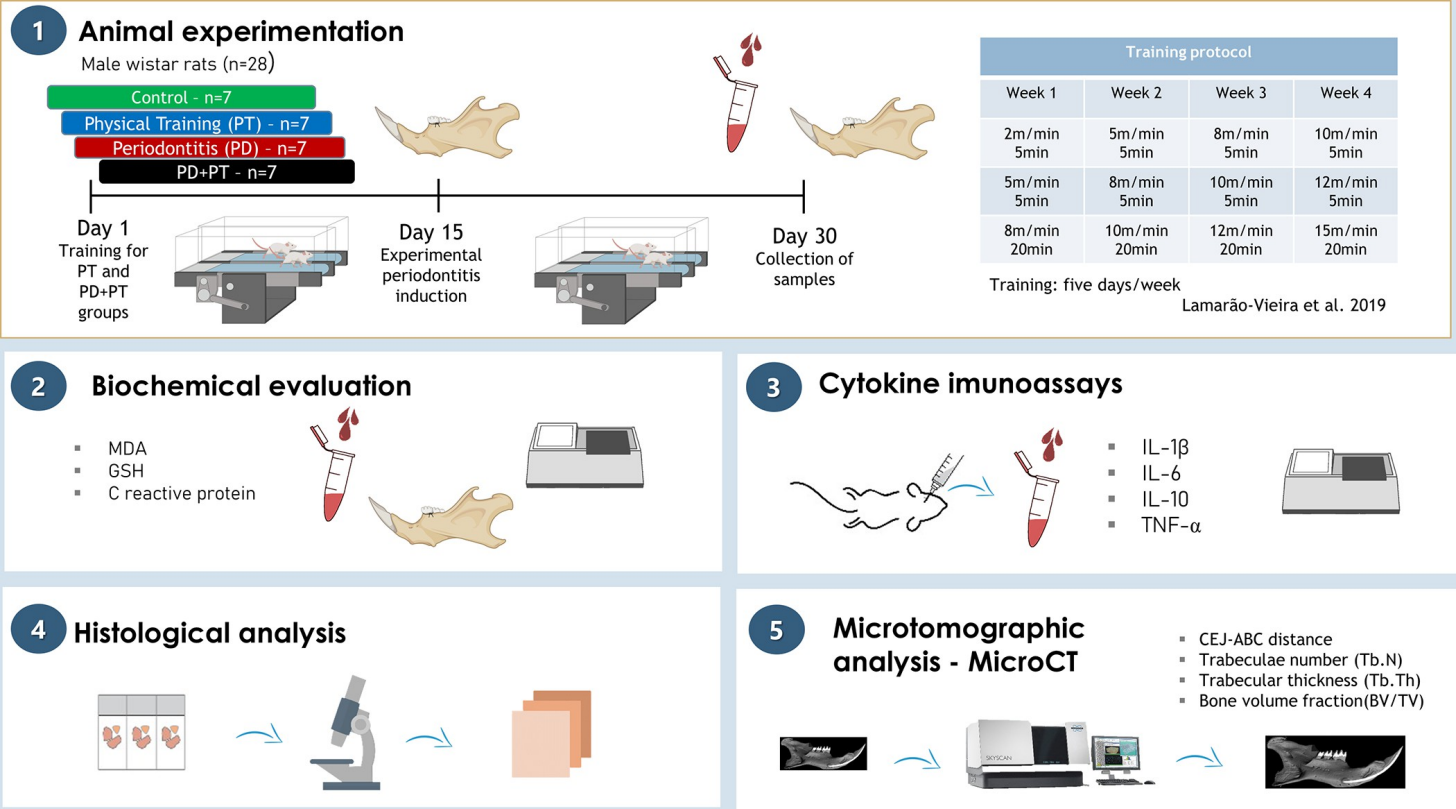
The methodological figure of the experimental steps. In (1), the animal experimental procedures, training protocol, and the sample collection stage are displayed; In (2), the biochemical evaluation is represented by the blood and tissue samples; In (3), plasma collection samples and microplate reader figures represented the cytokine assays; In (4), the histopathological evaluation is represented by the light field microscopy schematic, and in (5) the microtomographic analysis (micro-CT) is displayed. This figure was created with BioRender.com, accessed on 14 December 2022.

Sample size calculation was performed from the primary endpoint related to the level of interleukin-1beta of a previous study (SD 0.10; Power 95%, α = 5%) [10]. A number of 28 animals was considered sufficient for the four experimental groups. Male *Rattus novergicus* (n = 28; 90–120 days old, 150g-200g) were acclimated during 5 days in polypropylene cages in groups of 4 animals. Aseptic conditions, with controlled food (NUVITAL) and water *ad libitum*, 12 hours light/dark cycle and controlled temperature (25±1°C) were provided. During the final two days of acclimation, rodents were submitted to a trainability test protocol in the treadmill [17] to perform a block randomization. Two blinded examiners assessed the trainability of animals and randomly divided in 2x2 factorial arrangement (presence/absence of physical training and presence/absence of periodontitis) (weighted kappa: 0.7691): (Gl–Non-trained / G2: PD / G3: Trained / G4: Trained+PD). The sample collection description and method steps are summarized in Fig 1.

### Physical training

A five-lane motorized treadmill with an adjustable plane set at 0° was used to carry out the exercise training (MASTER-ONE®, Ribeirão Preto, SP, Brazil). The treadmill encasing, separating the lanes, with front and back wall air holes for each lane, enabled uncontrolled airflow inside it. For each lane, the monitor on the treadmill controller displayed the number of electric shocks generated, the accumulated time of electric shocks, and the calculated distance considering the set velocity and the time lapsed, deducting the time spent receiving a shock.

The exercise protocol of Trained and Trained+PD animals consisted of treadmill running, 5 days per week, 30 minutes/per day, with progressive speed (2m/min until 15m/min) (Lamarão-Vieira et al., 2019) for 4 weeks (28 days). Speed adjustments were increased weekly and affected collectively all five lanes of the treadmill belt. All weeks follow the protocol of 2 warm-ups of 5 minutes at the lower speeds and a bout of 20 minutes at the faster speed of the week. The workload of training with a progressive speed increase was based on the maximum treadmill speed (16m/min). Initially, we trained the animals with 12,5%, 30%, and 53% of maximum treadmill speed on the first week (mild-intensity). Then, 30%, 53%, and 66,6% on the second week(mild-intensity). Also, we trained with 53%, 66,6%, and 75% on the third week(moderate-intensity). And finally, 66,6%, 75%, and 93,7% of maximum treadmill speed(moderate-intensity). Considering previous studies [18,19], two weeks the animals perform mild-intensity training followed by two weeks of moderate-intensity training.

An electric shock stimulation of 0.2–mA was discharged when the animal contacted the metal grids behind the back end of the treadmill belt. Furthermore, the experimenters encouraged the rats to run with a light push or a sound to minimize the experience of excessive electric shocks, especially during the first days of habituation to exercise on the running treadmill.

### Ligature-induced periodontitis protocol

In the final 14 days of the experiment, the animal groups PD and Trained+PD were submitted to periodontitis induction protocol by ligature in the lower first molars. The rodents were anesthetized with ketamine hydrochloride solution (70mg/kg) and xylazine hydrochloride solution (7mg/kg) and after the absence of corneal reflexes, a cotton thread was positioned around the marginal gingiva with the aid of a Castroviejo needle holder and a Dietrich clamp (Quinelato®, Rio Claro, SP, Brazil). The researchers checked the ligatures twice a week. If the ligature was not in place, it was reinserted.

### Euthanasia and collection of samples

After the experimental period, the animals were euthanized by exsanguination under anesthesia (ketamine and xylazine hydrochloride solution, 300mg/kg and 30mg/kg, respectively) for collection of blood samples in K3 EDTA tubes and serum separating tubes, gingival tissue, and mandibles for morphological and biochemical analyzes. The animals were submitted to formalin solution perfusion to allow post-fixation of samples for morphological assessment. Blood samples were centrifuged at 3500 rpm (~1500G) for 10 minutes and plasma was stored at -80°C [20]. Right hemimandibles were dissected and gingival tissue was immediately stored at -80°C to maintain their properties for biochemical analyses. The hemimandible bone was stored in formaldehyde 10% for further microtomography evaluation. Post-fixed left hemimandibles were decalcified in a solution of EDTA 10% for 45 days. After decalcification, specimens were dehydrated in increasing ethanol concentrations, diaphanized in xylene, and embedded in paraffin.

### High-sensitivity C-reactive protein assessment

The hs-CRP concentration was determined by Immunoturbidimetric assay on an automated analyzer using reagents from Diasorin (Stillwater, Minnesota). The standard samples coated with antirat CRP antibody aggregates with the serum CRP, forming immune complexes. The formed immune complexes caused an increase in turbidity measured at 572 nm, which is proportional to the concentration of CRP in blood. The high-sensitivity C-reactive protein concentration was determined from CRP standards of known concentration.

### Malondialdehyde Dosage (MDA)

The content of MDA a product of lipid peroxidation, in the gingival samples (n = 7/group) was measured by the assay previously described [21]. Samples were suspended in Tris 1:5 (w/v) buffer. The material was incubated for 40 minutes at 45°C in a water bath, and centrifuged at 2500 G for 5 minutes at 4°C; 300 μL was then removed, read at 586 nm, and interpolated in a standard curve. Supernatants were tested for MDA content and placed in microplates. The absorbance of each sample was measured at 586 nm. The results are expressed as nanomoles of MDA per ml of plasma or mg of tissue.

### Glutathione Dosage (GSH)

GSH levels were measured to verify antioxidant activity [22]. Briefly, 0.02 M EDTA was added to the prepared tissue, 7/per group, and stored at -80°C until use. For GSH determination, the samples were thawed and automatically homogenized for 2 min. Samples were then centrifuged at 3000xg for 15 min at 4°C, then the supernatant (400 μl) was removed, and 800 μl of 0.4 M Tris buffer (pH 8.9) and 20 μl of 5,5’-dithiobis-(2-nitrobenzoic acid) were added. Absorbance was measured at 420 nm, and results were reported as mg of GSH units per mg of tissue.

### Cytokine immunoassays

Samples of plasma (n = 7/group) were processed [23]. Briefly, interleukin (IL)-1β (detection range, 62.5–4000 pg/mL; minimum detection limit, 12.5 ng/mL), IL-6 (detection range:125–8000 pg/mL) IL-10 (detection range: 62.5–4000 pg/mL; minimum detection limit, 12.5 ng/mL) and tumor necrosis factor (TNF)-α (detection range, 62.5–4000 pg/mL; minimum detection limit, 50 ng/ml) levels were determined by commercial enzyme-linked immunosorbent assay kits (R & D Systems, Minneapolis, MN), according to the manufacturer’s instructions. The results were expressed as pg/mL.

### Microtomographic analysis

Using micro-computed tomography (MicroCT.SMX-90 CT; Shimadzu Corp., Kyoto, Japan), the impact of exercise on periodontitis in the alveolar bone tissue was assessed in the right hemimandible. Samples were inserted into the device, and 360-degree rotation images were captured at 70 kV and 100 mA of intensity. Subsequently, pictures were reconstructed using the inspeXio SMX-90CT software (Shimadzu Corp., Kyoto, Japan) at a resolution of 1024 × 1024 and a thickness of 14 μm. The result generated 541 images for each sample.

The bone pictures were collected at the interradicular region, close to the furcation zone of the mandibular first molar. The region of interest (ROI) was defined as the interradicular region of the mandibular first molar from the apical third to the cervical third, which has an average area of 0.200 mm^2^. A threshold was used to distinguish the different gray levels in the image. Furthermore, a single-blind examiner conducted the measurements using the software application ImageJ (National Institutes of Health, Bethesda, Maryland, USA). The variations in gray levels of bone and other structures in images were used to determine the threshold. Based on this, the threshold was increased from 120 to 255. Trabecular thickness (Tb.Th), trabecular separation (Tb.Sp), and bone volume (BV/TV) were all quantified using the BoneJ plugin (Souza-Monteiro et al., 2021).

RadiAnt DICOM Viewer 5.0.1 (Medicant, Poznan, Poland) was employed for the three-dimensional (3D) reconstruction of the right hemimandible. The 3D images of the samples were placed in a standard position, allowing the buccal and lingual surfaces of the teeth to be examined. The distance between the cementoenamel junction and the alveolar bone crest was chosen as an evaluation metric to assess the potential impact on bone loss. Bone loss was identified by measuring the distance between the cementoenamel junction and the alveolar bone crest at six places on the mandibular first molar (i.e., mesial-buccal, buccal-medial, disto-buccal, mesial-lingual, lingual-medial, distolingual) and averaging these positions.

### Histological analysis

The left hemimandibles were collected and fixed in 4% formaldehyde for 24 hours. Subsequently, they were washed for 12 hours in running water and demineralized in 10% ethylenediaminetetraacetic acid (EDTA) for 45 days, with a weekly change of liquid and. After this period, specimens were washed for 24 hours in running water. Then, the samples were dehydrated in alcohol, diaphanized in xylene and embedded in paraffin. After inclusion, sections with a thickness of 5 μm were placed on individual slides stained with Hematoxylin and Eosin (HE) for observation under optical microscopy. To perform these analyses, images were obtained by a color digital camera (Cyber Shot DSC W-230, 4X optical zoom, Sony, Tokyo, Japan) coupled to a microscope (1.5x, Eclipse E200, Nikon, Tokyo, Japan; at 40X magnification). Captured images were imported to Image J software (NIMH, NIH, Bethesda, MD, United States).

### Statistical analysis

ANOVA one-way followed by Tukey post-hoc test was employed. Additionally, for bone loss mitigation, cytokine and oxidative stress assessment, a multivariate analysis of variance (MANOVA) was conducted to encompass all parameters. Moreover, a Pearson’ correlation coefficient compared variables addressing their relationships. Results were presented as mean ± standard error of the mean (SEM), and statistical significance was determined at p<0.05. Statistical analysis were performed using GraphPad Prism 8.0 software (Graphpad, San Diego, CA, USA) and Jamovi version 2.3.38 (The jamovi project (2024).[Computer Software]. Retrieved from https://www.jamovi.org).

## Results

### Aerobic physical training effects on systemic inflammatory response in induced periodontitis

**Fig 2:**
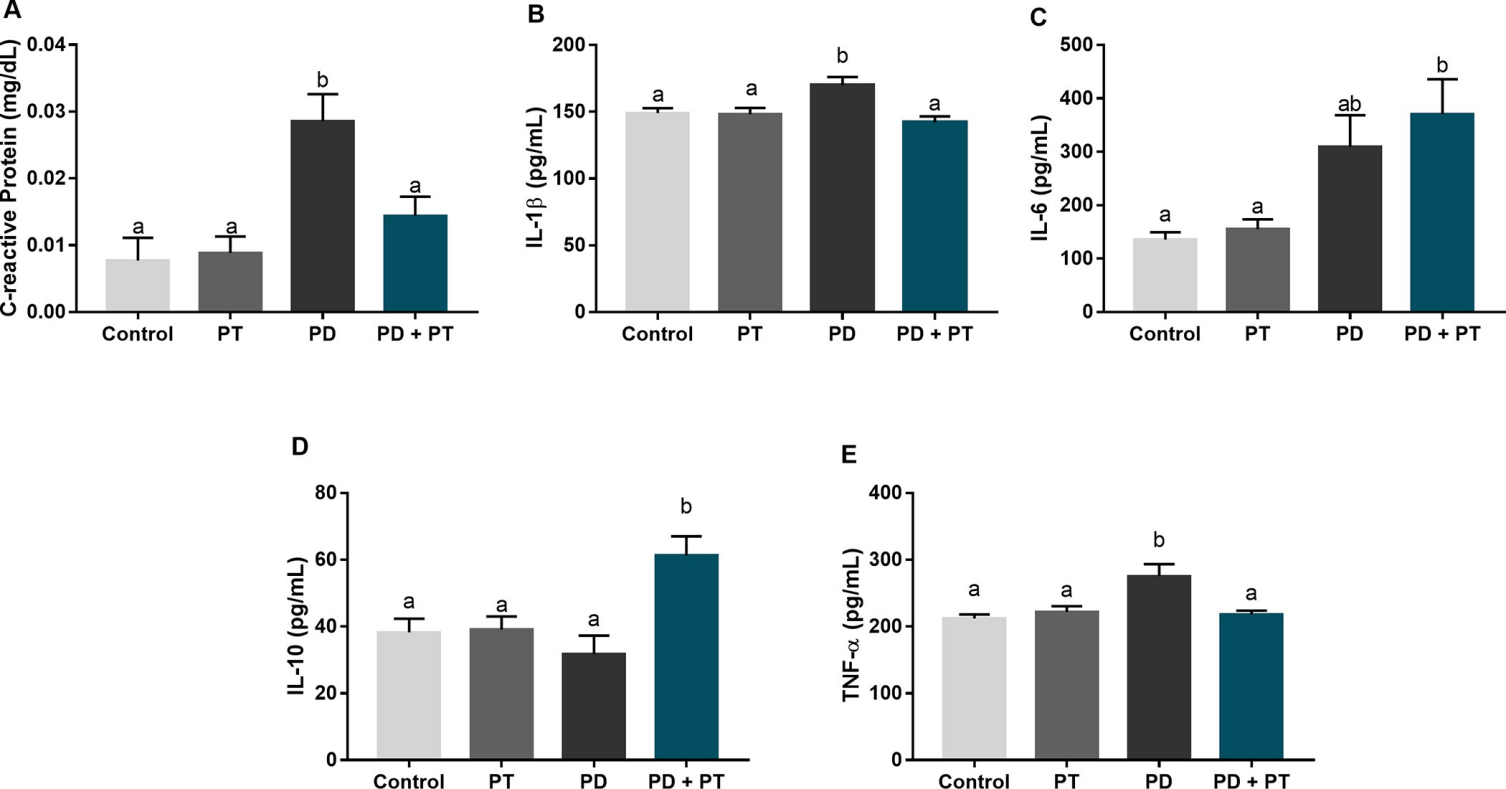
Systemic inflammatory biomarker levels of rats submitted or not to aerobic physical training and experimental-induced periodontitis. In A, C-reactive Protein (mg/dL); In B, Interleukin-1β (IL-1β; pg/mL); In C, Interleukin-6 (IL-6; pg/mL); In D, Interleukin-10 (IL-10; pg/mL); and in E, Tumoral Necrosis Factor α (TNF-α; pg/mL). Similar letters indicate no statistical difference (p>0.05) and different superscript letters indicate statistical difference (p<0.05) by ANOVA-1 way with Tukey’s post hoc test. In CRP, IL-1β, and TNF- α analyses, the difference was only detected in the PD group. While IL-10 and IL-6, only PD and PD+PT show statistical differences.

Considering the weight and the distance performed by animals, no significant differences were observed (p = 0.1603; and p = 0.1087, respectively). The systemic inflammatory profile featured in induced periodontitis was modulated by aerobic physical training in rats. In Fig 2A, CRP was significantly increased in the group only with experimental-induced periodontitis in comparison to the control (p = 0.0007). On the other hand, trained animals with periodontitis showed similar levels of CRP in comparison to the control group, with no statistically significant difference (p = 0.4745). In Fig 2B, the IL-1β levels were also increased in the periodontitis group when compared to the control (p = 0.0365) but were restored to control levels when associated with physical training (p = 0.7473).

In contrast, in Fig 2C, physical training did not decrease IL-6 levels in animals with periodontitis in comparison to the periodontitis group (p = 0.1051), and changes were observed in IL-10 levels trained animals with periodontitis (p = 0.0186; Fig 2D). TNF-α levels were restored to control levels with the physical training (p = 0.978; Fig 2E).

### Oxidative stress triggered by periodontitis is modulated by aerobic physical training

**Fig 3:**
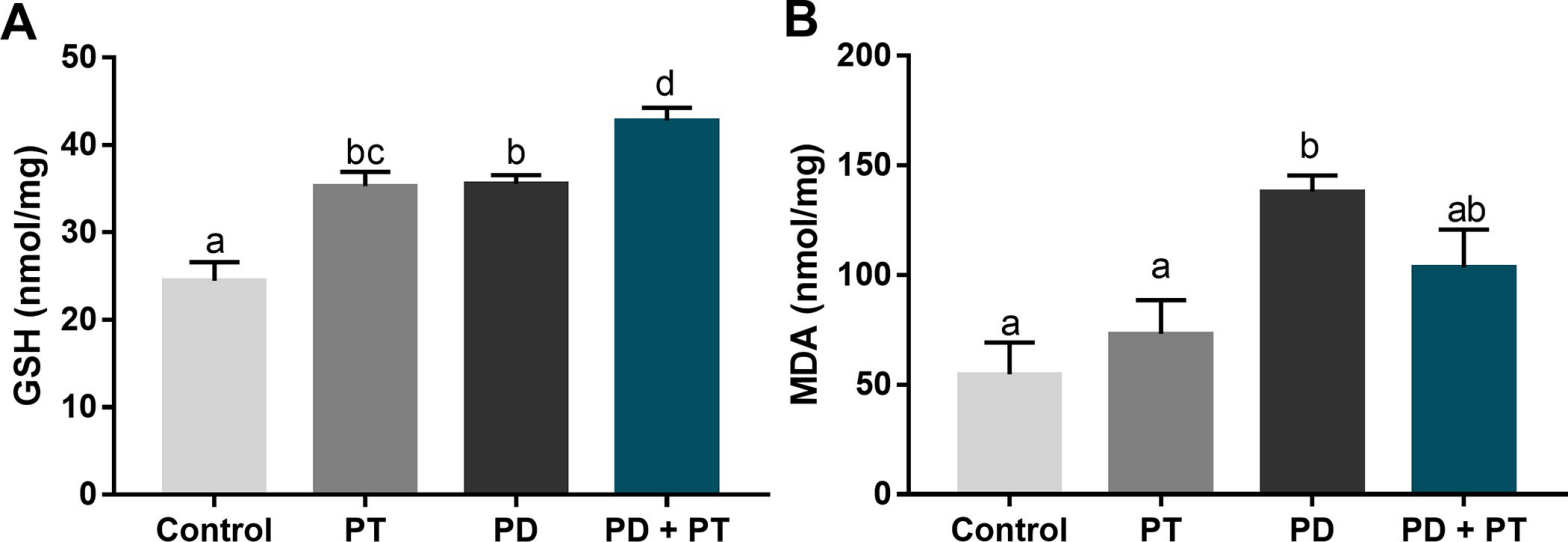
Local oxidative biochemistry markers levels of rats submitted or not to aerobic physical training and experimental-induced periodontitis. In A, Reduced Glutathione (GSH; nmol/mg), and in B, Lipid Peroxidation determined by Malondialdehyde levels (MDA; nmol/mg). Different superscript letters indicate statistical difference (p<0.05) by ANOVA-1 way with Tukey’s post hoc test.

The reduced glutathione levels were increased in the presence of periodontal disease, as shown in Fig 3A. Analyzing the interventions solely, both induced periodontitis (p = 0.0002) and physical training (p = 0.0002) increased their antioxidant levels in comparison to the control group. On the other hand, the diseased and trained group showed reduced glutathione levels over increased in comparison to all the groups (p<0.05). Regarding lipid peroxidation levels (Fig 3B), they were significantly increased in the presence of periodontitis (p = 0.0059), but when physical training was combined with the experimental-induced periodontitis, no statistically significant difference was observed in comparison to control group (p = 0.1188).

### Histological damage in alveolar bone is minimized in trained rats with induced periodontitis

**Fig 4:**
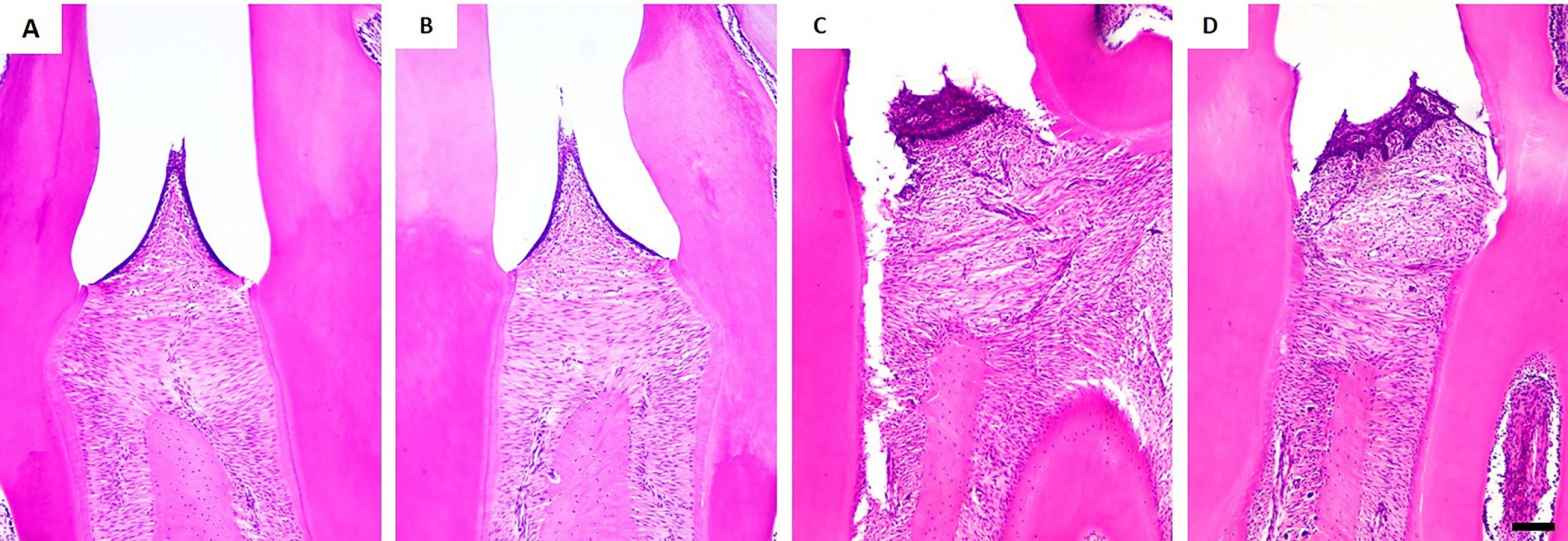
Gross histopathology of the periodontium of rats submitted or not to aerobic physical training and experimental-induced periodontitis. Letters A, B, C, and D represent Control, Physical Training, Periodontitis, and Periodontitis + Physical Training groups, respectively. Magnification of 10x. Scale bar: 200μm.

The gross histopathological analysis of rats’ periodontium (Fig 4) showed important changes present in periodontitis that are minimized by aerobic physical training. In column A, the control group showed the preserved epithelial and connective tissues. Similar tissue integrity was observed in physical training group (Fig 4B). Eosinophilic horizontal structures evidenced are collagen fibers connecting the tooth mineralized surface and the gum epithelium, which was severely damaged by experimentally-induced periodontitis shown in column C, with the remarked presence of polymorphonuclear cells in remaining connective tissue without collagen fibers structures (Fig 4C). On the other hand, aerobic physical training (Fig 4D) reduced inflammatory infiltration and preserved most of the collagen fibers in Periodontitis + Physical Training group, as highlighted in Fig 4D.

### Morphological and microstructural impairments caused by periodontitis are attenuated by aerobic physical training

**Fig 5:**
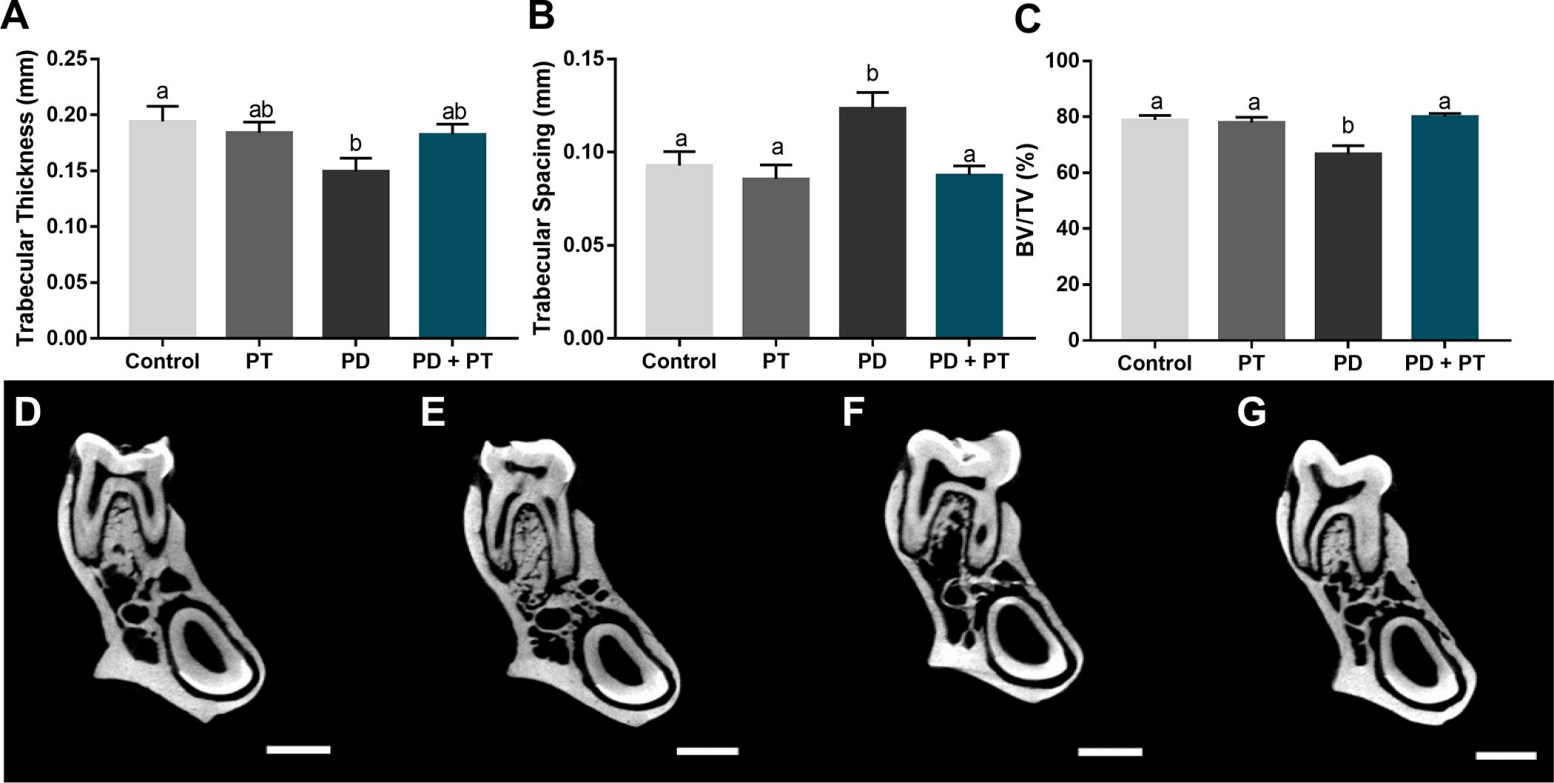
Alveolar bone microtomographic assessment in rats submitted or not to aerobic physical training and experimental-induced periodontitis. In A, Trabecular Thickness (mm); In B, Trabecular Spacing (mm); In C, the ratio between Bone Volume (BV) and Tissue Volume (TV) expressed as a percentage (%). Different superscript letters indicate statistical difference (p<0.05) by ANOVA-1 way with Tukey’s post hoc test. Microtomographic images D, E, F and G, illustrate the inter radicular alveolar bone of groups Control, Physical Training (PT), Periodontitis (PD), and Periodontitis + Physical Training (PD + PT), respectively. Scale bar: 1 mm.

Induced periodontitis affected the alveolar bone quality as seen in Fig 5. The Tb.Th was significantly reduced in comparison to the control group (p = 0.0443), which was minimized by the occurrence of physical training (Fig 5A). In the same perspective, the Tb.Sp was significantly increased in periodontitis group in comparison to the control (Fig 5B; p = 0.0301), but the trained group with periodontitis did not differ from controls Tb.Sp (p = 0.9606). The BV/TV ratio was reduced with periodontitis *per se* in comparison to the control group (p = 0.0016, Fig 5C), but it was kept the same as the control in the periodontitis plus physical training group (p = 0.9804). Letters D, E, F, and G represents Control, Physical Training (PT), Periodontitis (PD), and Periodontitis + Physical Training (PD + PT), respectively. Scale bar: 1 mm.

**Fig 6:**
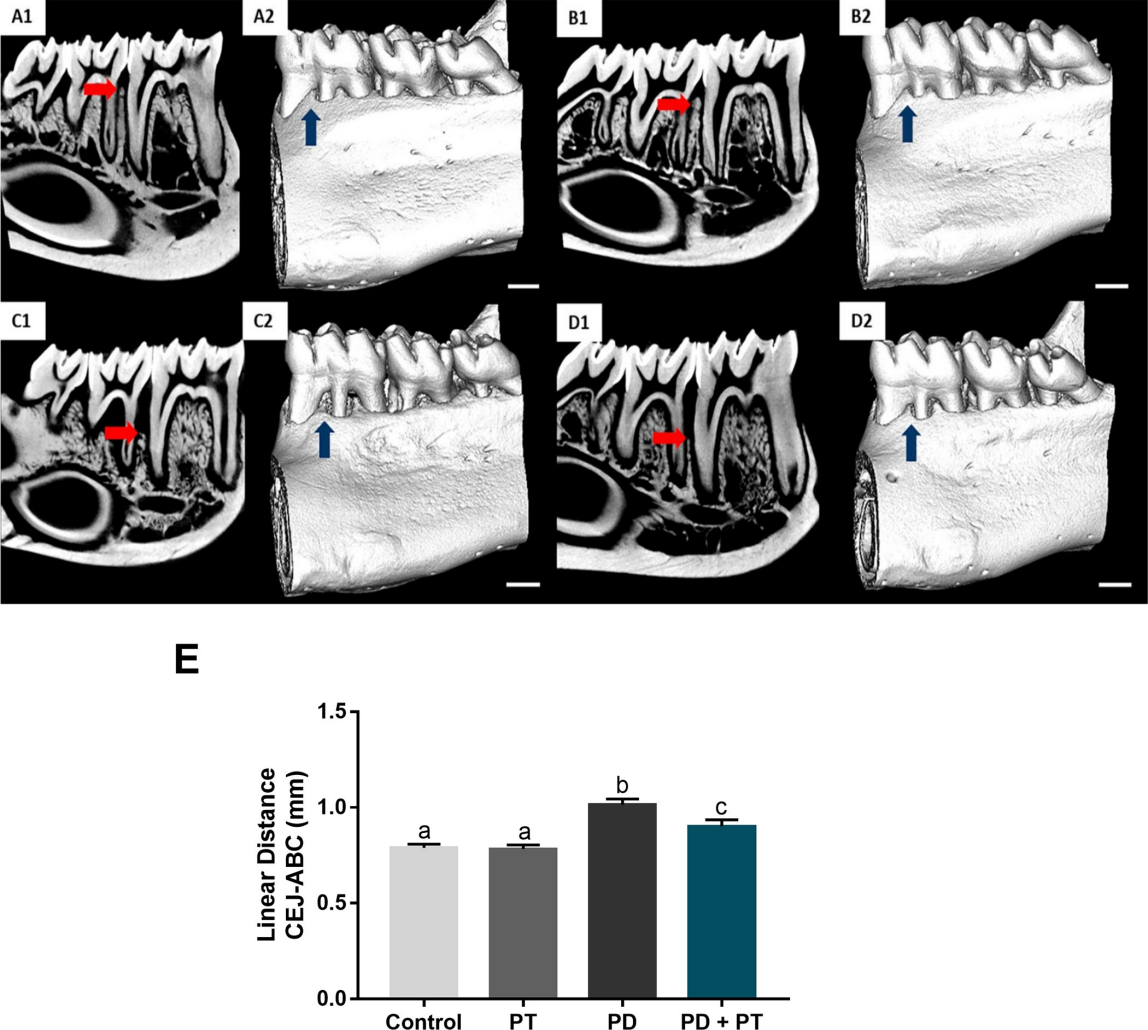
Alveolar bone loss analysis of rats submitted or not to aerobic physical training and experimental-induced periodontitis. Red arrows A1, B1, C1, and D1 shows alveolar bone crest loss in Control, Physical Training (PT), Periodontitis (PD), and Periodontitis + Physical Training (PD + PT), respectively. Microtomographic images B2, C2, D2, and D2, illustrate the quantitative results in E of groups Control, Physical Training (PT), Periodontitis (PD), and Periodontitis + Physical Training (PD + PT), respectively. Blue arrows highlight alveolar bone loss in buccal region between molar roots. In E, the measurement of the linear distance (mm) between the cementum-enamel junction (CEJ) and alveolar bone crest (ABC). Different superscript letters indicate statistical difference (p<0.05) by ANOVA-1 way with Tukey’s post hoc test. Scale bar: 1 mm.

In addition to alveolar bone quality demonstrated in Fig 3, physical training was also able of modulating the alveolar bone dimension loss in periodontitis. Periodontitis *per se* (Fig 6C) significantly increased (p <0.0001) the linear distance between the cementum-enamel junction and alveolar bone crest in comparison to the control group (Fig 6B).The presence of aerobic physical training (Fig 6E) did not restore vertical loss to basal levels; however, it was able to minimize such morphological damage caused by periodontitis (Fig 6C) (p = 0.0337).

The multivariate analysis of variance (MANOVA) conducted a regression analysis for bone loss mitigation, cytokines, and oxidative stress variables (shown in Table 1).

**Table 1:** MANOVA analysis for bone loss mitigation, cytokine and biochemical variables using Pillai’ Trace test.

Multivariate test–Bone loss mitigation													
				Value		F		df1		df2		p	
Groups All dependent variables		Pillai’ Trace		2.75		3.99		33		12		0.007	
Groups Cytokine and oxidative stress		Pillai’ Trace		2.33		4.00		21		24		< .001	

df1: Degrees of freedom related to the groups being compared for bone loss mitigation, cytokine and biochemical variables.

df2: Degrees of freedom associated with the error term.

Furthermore, Pearson’ correlation coefficient was included. The strongest correlation observed was between MDA and C reactive protein (0.703; p<0.001). Moderate correlations were found between the following pairs: BV/TV and Tb.Sp (-0.668; p<0.001); CRP and IL-1β (0.648; p<0.001); CRP and Tb.Sp (0.648; p<0.001); BV/TV and TNF- α (-0.635; p<0.001); ABC-CEJ and MDA (0.629; p<0.001); IL-1β and TNF- α (0.624; p<0.001); CRP and TNF-α (0.623; p<0.001); CRP and BV/TV(-0.620; p<0.001); Tb.Th and ABC-CEJ (-0.616; p<0.001); Tb.Sp and IL-1β (0.612; p<0.001); BV/TV and Tb.Th (-0.596; p<0.001); IL-1β and MDA (0.562; p = 0.002); Tb.Sp and TNF- α (0.540; p = 0.003); CRP and ABC-CEJ (0.534; p = 0.003); BV/TV and ABC-CEJ (0.512; p = 0.005); BV/TV and MDA (-0.502; p = 0.007); BV/TV and IL-1β (-0.500; p = 0.007). For a comprehensive overview of the correlation between the variables, please refer to supplementary materials.

## Discussion

In the present study, aerobic physical training was able to attenuate alveolar bone loss and also reduced the systemic pro-inflammatory markers and local oxidative stress levels in ligature-induced periodontitis in rats. To the best of our knowledge, this is the first experimental study to bring evidence of the structured aerobic physical training in the attenuation of periodontitis and to show multiple pathogenic mechanisms targeted by the exercise. Considering the intrinsic role of oxidative stress and inflammatory cascades in the establishment and progression of periodontal diseases, physical exercise significantly mitigates such detrimental effects leading in better histological and microstructural tissue quality, reducing the impacts on alveolar bone loss.

Considering the methodological variables of exercise, authors report that intensity and frequency are related to increased physical capacity [2]. Regular aerobic exercise of 150 to 300 minutes per week implies a reduction in insulin resistance, an increase in glucose consumption by skeletal muscles and a 0.5–0.7% reduction in Hb1Ac levels [3,16], and a reduction in the prevalence of periodontitis [13]. The present study used a validated physical exercise experimental model with rodents, who run on a treadmill. Such model has the advantage of calibrating the intensity and duration of the exercise, facilitating the characterization of the exercise, whether of low, moderate and high intensity or short, intermittent or long-term [24]. When comparing our results with studies that applied forced swimming protocol [14,15] associated with a ligature-induced periodontitis model, the reductions in inflammatory markers and bone loss were similar. In this way, the treadmill running protocol showed significant results with lower intensity than the forced swimming protocol.

The kinetics involving the process of ligature-induced periodontitis was previously described [25–27]. After insertion of the cotton, silk or nylon thread in rat molars (upper or lower molars], alveolar bone loss can be observed through radiographic and histologic methods, from the 3rd to 7th day of insertion of the ligature and bone loss. The progression of bone loss extends to the 15th day, with no significant differences from the 21st day in upper molars and 30th day onwards in lower molars [25]. Thus, the time of injury covered in this study includes an appropriate period of evaluation of periodontal breakdown [25].

Periodontal tissue loss is marked by oxidative stress [28]. Superoxide radicals and its sub-products such as MDA are observed in crevicular fluid of patients with periodontitis. Their presence is related to the role of immune systems cells, mainly PMNs, lymphocytes, and osteoclasts [29,30]. Although regular exercise protects against many diseases, it is also linked to cell and tissue damage by exercise-induced oxidative stress [31,32]. It is important to emphasize that the reactive species during the practice of physical exercises can be produced by different mechanisms as partial reduction of oxygen in the mitochondria, inflammatory process, as well as the processes of ischemia and reperfusion [32,33]. Nevertheless, studies also suggest that physical exercise can cause antioxidant parameters increase (GSH, catalase, SOD, uric acid) in a dose-response pattern providing a resistance against stress [31,33].

Among non-enzymatic antioxidants, reduced glutathione is one of the most important low-weight molecules in reducing the levels of hydroxyl radicals in the periodontium [34,35]. In addition, GSH plays an important role in the intracellular oxidative balance in the regulation of cellular signals and gene transcription factors, such as the nuclear factor signaling pathway–κβ (NF-κβ) [14,34]. The down-regulation of NF-κB and its target genes, which actively participate in bone resorption processes, in addition to inhibiting the activity and differentiation of osteoclasts, acts on the activity of RANK-L, modulating inflammatory cytokines, decreasing the secretion of IL- 1α, IL-6 and TNF-α and increasing the secretion of IL-3, IL-4, IL-14 and IFNγ [35,36]. Therefore, the reduction of essential parameters in the bone resorption process may possibly be associated with the increase in GSH, which indicates a reduction in TNF-α expression; IL-1β and IL-6 [28,36].

Periodontal inflammation is a complex interaction of biomarkers against the dysbiotic biofilm. Without periodontal preventive and therapeutic intervention, interleukins (IL-1beta, IL-6, TNF-alpha, IL-10, etc.), plasma proteins (such as c-reactive protein) and oxidative stress markers generate a disproportionate immune and tissue response leading to connective tissue degradation and bone damage [35]. IL-1β and IL-6 are important interleukins with a role in cell migration and osteoclastogenesis. Both interleukins are influenced by the diverse effects of TNFαwhich upregulates the production of MMP-8 and RANKL characteristic of collagen fiber degradation and alveolar bone resorption in periodontitis[35]. Increased levels of these cytokines were found in intense exercise with predominant eccentric movements but not after moderate exercise [36]. Several studies demonstrate reductions in periodontal tissue interleukins levels in association with exercise of moderate intensity [15]. Thus, the inflammatory mediators shared by PD and moderate-intensity exercise could be related to reduction of periodontal tissue destruction [16].

Histological changes in ligature-induced periodontitis have been demonstrated and evidence points that they are set after 1 to 5 days, with increase of inflammatory infiltrate [21]. Recruitment of PMNs and also T and B lymphocytes to the infection site remains until 14 days and after 21 days in a chronic phase [35]. Also, the multiplication of specific periodontal pathogens tends to increase and develop a deeper inflammation in relation to the supporting tissues leading to collagen fiber loss of attachment [30]. Antibodies, plasmocytes, PMNs are the main cells found at this stage and responsible for the release of metalloproteinases (MMP-2 and MMP-9), reactive oxygen species, prostaglandins, cytokines (IL-1β, IL-6, TNF-α, protein C reactive) in dental supporting tissues [36]. According to this, the results showed by non-trained animals with periodontitis group were similar to results demonstrated in other studies [14], but trained animals with periodontitis showed reduced cell infiltrate as well as lower connective tissue breakdown and bone resorption.

Micro-computed tomography provides accurate volumetric assessments of cortical and trabecular bone while being non-destructive, and a short processing time analysis [26]. Rodent alveolar bone is investigated for a broad range of purposes, including developing knowledge of hereditary and acquired bone abnormalities, treatments to improve the quality or amount of bone, orthodontic tooth movement, and aging [26,37]. The degradation of cementum, periodontal ligament, and alveolar bone as well as tooth loss brought on by periodontitis have a major impact on oral and general health as well as quality of life. Therefore, the use of μCT to examine periodontal pathology, healing, and regeneration is quite useful and are considered contemporarily the “gold standard”. Linear distances between cementum-enamel junction to alveolar bone crest (CEJ-ABC) is found increased in ligature induced periodontitis [26]. However, changes may occur when exercise is applied. Stereomicroscopy identified changes in alveolar bone loss in rats submitted to exercise protocols [14,15]. Similar to previous results, this is the first study that identified reduced CEJ-ABC in trained rats with PD by Micro-CT.

Some limitations of this study should be highlighted. Although the direct assessment of cytokines in plasma reflects the low-level systemic inflammation of periodontitis, direct analysis of cytokines in periodontal tissue is important. This is due to the large systemic variations of inflammatory cytokines due to other conditions such as cardiovascular diseases, rheumatic diseases, smoking which, in studies with multivariate analyzes, may act as confounding factors in the evaluation of exercise and its effects on periodontal disease [12,38,39]. Another limitation is regarded to effective evaluation of training intensity by oxygen uptake (VO2 consumption) [24]. Metabolic chambers associated to treadmills can precisely evaluate exercise intensity proposed to training protocol, as well as they to establish whether exercise intensity is sufficient to produce clinical effects on dental supporting tissues. Also, it should be highlighted that translation of results from animal studies to clinical practice should be performed with caution. Nevertheless, the purpose of this study is to identify pre-clinical parameters with support further exercise researches in murine models or patients with periodontal disease.

On the other hand, it should be highlighted that the results encountered herein are in line with the associations demonstrated in epidemiological studies and the possible mechanisms involved in the role of exercise in preventing/modifying periodontal breakdown are plausible. In this sense, these results shed light into the fact that periodontal diseases are non-communicable chronic diseases that share risk factors with other diseases/conditions classified as such, suggesting adoption of healthy lifestyle in their management.

## Conclusion

Treadmill aerobic training minimized alveolar bone loss, reduced systemic inflammatory cytokine pattern, and increased local antioxidant parameters levels in ligature-induced periodontitis in rats. A significant relationship of oxidative stress changes and bone sorption cytokines appears to be a mechanism of inflammation balance by exercise. Regular frequency and moderate intensity may be related to the effects of training on tooth-supporting tissues.

## Supporting information

S1 File (DOCX)
