# Supplementary material for: Physical training minimizes immunological dysfunction, oxidative stress and tissue destruction on experimental periodontitis in rats
Source: PLoS One. 2024 Jun 6;19(6):e0303374. doi: 10.1371/journal.pone.0303374 (PMC11156300; doi:10.1371/journal.pone.0303374)
Supplement: S1 File — (DOCX) [file pone.0303374.s001.docx]

| Table 1. Correlation matrix of all data | | | | | | | | | | | | | | | | | | | | | | | | | |
| --- | --- | --- | --- | --- | --- | --- | --- | --- | --- | --- | --- | --- | --- | --- | --- | --- | --- | --- | --- | --- | --- | --- | --- | --- | --- |
|  | |  | | **IL-1β** | | **TNF-α** | | **IL-6** | | **IL-10** | | **GSH** | | **MDA** | | **CEJ-ABC distance** | | **Tb.Th** | | **Tb.Sp** | | **BV/TV** | | **C reactive Protein** | |
| IL-1β |  | Pearson’ R |  | — |  |  |  |  |  |  |  |  |  |  |  |  |  |  |  |  |  |  |  |  |  |
|  |  | df |  | — |  |  |  |  |  |  |  |  |  |  |  |  |  |  |  |  |  |  |  |  |  |
|  |  | p-value |  | — |  |  |  |  |  |  |  |  |  |  |  |  |  |  |  |  |  |  |  |  |  |
| TNF-α |  | Pearson’ R |  | 0.624 | *** | — |  |  |  |  |  |  |  |  |  |  |  |  |  |  |  |  |  |  |  |
|  |  | df |  | 26 |  | — |  |  |  |  |  |  |  |  |  |  |  |  |  |  |  |  |  |  |  |
|  |  | p-value |  | < .001 |  | — |  |  |  |  |  |  |  |  |  |  |  |  |  |  |  |  |  |  |  |
| IL-6 |  | Pearson’ R |  | 0.068 |  | 0.093 |  | — |  |  |  |  |  |  |  |  |  |  |  |  |  |  |  |  |  |
|  |  | df |  | 26 |  | 26 |  | — |  |  |  |  |  |  |  |  |  |  |  |  |  |  |  |  |  |
|  |  | p-value |  | 0.731 |  | 0.638 |  | — |  |  |  |  |  |  |  |  |  |  |  |  |  |  |  |  |  |
| IL-10 |  | Pearson’ R |  | -0.318 |  | -0.318 |  | 0.038 |  | — |  |  |  |  |  |  |  |  |  |  |  |  |  |  |  |
|  |  | df |  | 26 |  | 26 |  | 26 |  | — |  |  |  |  |  |  |  |  |  |  |  |  |  |  |  |
|  |  | p-value |  | 0.099 |  | 0.099 |  | 0.847 |  | — |  |  |  |  |  |  |  |  |  |  |  |  |  |  |  |
| GSH |  | Pearson’ R |  | -0.058 |  | 0.117 |  | 0.412 | * | 0.436 | * | — |  |  |  |  |  |  |  |  |  |  |  |  |  |
|  |  | df |  | 26 |  | 26 |  | 26 |  | 26 |  | — |  |  |  |  |  |  |  |  |  |  |  |  |  |
|  |  | p-value |  | 0.769 |  | 0.554 |  | 0.030 |  | 0.021 |  | — |  |  |  |  |  |  |  |  |  |  |  |  |  |
| MDA |  | Pearson’ R |  | 0.562 | ** | 0.397 | * | 0.348 |  | 0.131 |  | 0.308 |  | — |  |  |  |  |  |  |  |  |  |  |  |
|  |  | df |  | 26 |  | 26 |  | 26 |  | 26 |  | 26 |  | — |  |  |  |  |  |  |  |  |  |  |  |
|  |  | p-value |  | 0.002 |  | 0.037 |  | 0.070 |  | 0.506 |  | 0.111 |  | — |  |  |  |  |  |  |  |  |  |  |  |
| CEJ-ABC distance |  | Pearson’ R |  | 0.390 | * | 0.493 | ** | 0.351 |  | -0.045 |  | 0.283 |  | 0.629 | *** | — |  |  |  |  |  |  |  |  |  |
|  |  | df |  | 26 |  | 26 |  | 26 |  | 26 |  | 26 |  | 26 |  | — |  |  |  |  |  |  |  |  |  |
|  |  | p-value |  | 0.040 |  | 0.008 |  | 0.067 |  | 0.818 |  | 0.145 |  | < .001 |  | — |  |  |  |  |  |  |  |  |  |
| Tb.Th |  | Pearson’ R |  | -0.189 |  | -0.443 | * | -0.072 |  | 0.093 |  | -0.152 |  | -0.375 | * | -0.616 | *** | — |  |  |  |  |  |  |  |
|  |  | df |  | 26 |  | 26 |  | 26 |  | 26 |  | 26 |  | 26 |  | 26 |  | — |  |  |  |  |  |  |  |
|  |  | p-value |  | 0.336 |  | 0.018 |  | 0.714 |  | 0.638 |  | 0.441 |  | 0.049 |  | < .001 |  | — |  |  |  |  |  |  |  |
| Tb.Sp |  | Pearson’ R |  | 0.612 | *** | 0.540 | ** | 0.290 |  | -0.184 |  | 0.001 |  | 0.493 | ** | 0.345 |  | -0.090 |  | — |  |  |  |  |  |
|  |  | df |  | 26 |  | 26 |  | 26 |  | 26 |  | 26 |  | 26 |  | 26 |  | 26 |  | — |  |  |  |  |  |
|  |  | p-value |  | < .001 |  | 0.003 |  | 0.134 |  | 0.350 |  | 0.997 |  | 0.008 |  | 0.072 |  | 0.648 |  | — |  |  |  |  |  |
| BV/TV |  | Pearson’ R |  | -0.500 | ** | -0.635 | *** | -0.198 |  | 0.324 |  | -0.054 |  | -0.502 | ** | -0.512 | ** | 0.596 | *** | -0.668 | *** | — |  |  |  |
|  |  | df |  | 26 |  | 26 |  | 26 |  | 26 |  | 26 |  | 26 |  | 26 |  | 26 |  | 26 |  | — |  |  |  |
|  |  | p-value |  | 0.007 |  | < .001 |  | 0.313 |  | 0.092 |  | 0.786 |  | 0.007 |  | 0.005 |  | < .001 |  | < .001 |  | — |  |  |  |
| C reactive Protein |  | Pearson’ R |  | 0.648 | *** | 0.623 | *** | 0.321 |  | -0.118 |  | 0.297 |  | 0.703 | *** | 0.534 | ** | -0.238 |  | 0.648 | *** | -0.620 | *** | — |  |
|  |  | df |  | 26 |  | 26 |  | 26 |  | 26 |  | 26 |  | 26 |  | 26 |  | 26 |  | 26 |  | 26 |  | — |  |
|  |  | p-value |  | < .001 |  | < .001 |  | 0.096 |  | 0.551 |  | 0.124 |  | < .001 |  | 0.003 |  | 0.223 |  | < .001 |  | < .001 |  | — |  |
| Note. * p < .05, ** p < .01, *** p < .001 | | | | | | | | | | | | | | | | | | | | | | | | | |
|  | | | | | | | | | | | | | | | | | | | | | | | | | |

Table 2. ANOVA one-way analysis of variables

| CRP analysis |  |  |  |  |  |
| --- | --- | --- | --- | --- | --- |
|  |  |  |  |  |  |
| Tukey's multiple comparisons test | Mean Diff, | 95,00% CI of diff, | Significant? | Summary | Adjusted P Value |
| Control vs. PT | -0,001143 | -0,01380 to 0,01152 | No | ns | 0,9944 |
| Control vs. PD | -0,02086 | -0,03352 to -0,008195 | Yes | *** | 0,0007 |
| Control vs. PD + PT | -0,006714 | -0,01938 to 0,005948 | No | ns | 0,4745 |
| PT vs. PD | -0,01971 | -0,03238 to -0,007052 | Yes | ** | 0,0013 |
| PT vs. PD + PT | -0,005571 | -0,01823 to 0,007091 | No | ns | 0,6243 |
| PD vs. PD + PT | 0,01414 | 0,001481 to 0,02680 | Yes | * | 0,0246 |
|  |  |  |  |  |  |
| IL-1β analysis | | | | | |
| Tukey's multiple comparisons test | Mean Diff, | 95,00% CI of diff, | Significant? | Summary | Adjusted P Value |
| Control vs. PT | 1 | -18,87 to 20,87 | No | ns | 0,9989 |
| Control vs. PD | -21 | -40,87 to -1,127 | Yes | * | 0,0365 |
| Control vs. PD + PT | 7 | -12,87 to 26,87 | No | ns | 0,7473 |
| PT vs. PD | -22 | -41,87 to -2,127 | Yes | * | 0,0275 |
| PT vs. PD + PT | 6 | -13,87 to 25,87 | No | ns | 0,8232 |
| PD vs. PD + PT | 28 | 8,127 to 47,87 | Yes | ** | 0,0048 |
|  |  |  |  |  |  |
| IL-6 analysis | | | | | |
| Tukey's multiple comparisons test | Mean Diff, | 95,00% CI of diff, | Significant? | Summary | Adjusted P Value |
| Control vs. PT | -19,37 | -197,1 to 158,3 | No | ns | 0,9903 |
| Control vs. PD | -173,7 | -351,3 to 4,032 | No | ns | 0,0571 |
| Control vs. PD + PT | -234,9 | -412,6 to -57,20 | Yes | ** | 0,0066 |
| PT vs. PD | -154,3 | -332,0 to 23,40 | No | ns | 0,1051 |
| PT vs. PD + PT | -215,5 | -393,2 to -37,83 | Yes | * | 0,0134 |
| PD vs. PD + PT | -61,23 | -238,9 to 116,5 | No | ns | 0,7781 |
|  |  |  |  |  |  |
| IL-10 analysis | | | | | |
| Tukey's multiple comparisons test | Mean Diff, | 95,00% CI of diff, | Significant? | Summary | Adjusted P Value |
| Control vs. PT | -0,8214 | -19,93 to 18,28 | No | ns | 0,9994 |
| Control vs. PD | 6,551 | -12,55 to 25,66 | No | ns | 0,7806 |
| Control vs. PD + PT | -23,01 | -42,11 to -3,904 | Yes | * | 0,0141 |
| PT vs. PD | 7,373 | -11,73 to 26,48 | No | ns | 0,7138 |
| PT vs. PD + PT | -22,19 | -41,29 to -3,083 | Yes | * | 0,0186 |
| PD vs. PD + PT | -29,56 | -48,66 to -10,46 | Yes | ** | 0,0014 |
|  |  |  |  |  |  |
| TNF-α analysis | | | | | |
| Tukey's multiple comparisons test | Mean Diff, | 95,00% CI of diff, | Significant? | Summary | Adjusted P Value |
| Control vs. PT | -9,686 | -51,97 to 32,59 | No | ns | 0,9207 |
| Control vs. PD | -63,4 | -105,7 to -21,12 | Yes | ** | 0,002 |
| Control vs. PD + PT | -6,114 | -48,39 to 36,17 | No | ns | 0,978 |
| PT vs. PD | -53,71 | -95,99 to -11,43 | Yes | ** | 0,0092 |
| PT vs. PD + PT | 3,571 | -38,71 to 45,85 | No | ns | 0,9954 |
| PD vs. PD + PT | 57,29 | 15,01 to 99,57 | Yes | ** | 0,0053 |
|  |  |  |  |  |  |
| GSH analysis | | | | | |
| Tukey's multiple comparisons test | Mean Diff, | 95,00% CI of diff, | Significant? | Summary | Adjusted P Value |
| Control vs. PT | -10,83 | -16,99 to -4,675 | Yes | *** | 0,0002 |
| Control vs. PD | -11,09 | -17,25 to -4,934 | Yes | *** | 0,0002 |
| Control vs. PD + PT | -18,34 | -24,50 to -12,18 | Yes | **** | <0,0001 |
| PT vs. PD | -0,2593 | -6,418 to 5,900 | No | ns | 0,9995 |
| PT vs. PD + PT | -7,509 | -13,67 to -1,350 | Yes | * | 0,012 |
| PD vs. PD + PT | -7,25 | -13,41 to -1,091 | Yes | * | 0,016 |
|  |  |  |  |  |  |
| MDA analysis | | | | | |
| Tukey's multiple comparisons test | Mean Diff, | 95,00% CI of diff, | Significant? | Summary | Adjusted P Value |
| Control vs. PT | -18,52 | -77,54 to 40,50 | No | ns | 0,7889 |
| Control vs. PD | -83,33 | -142,4 to -24,31 | Yes | ** | 0,0059 |
| Control vs. PD + PT | -48,81 | -107,8 to 10,21 | No | ns | 0,1188 |
| PT vs. PD | -64,81 | -123,8 to -5,796 | Yes | * | 0,0301 |
| PT vs. PD + PT | -30,29 | -89,31 to 28,73 | No | ns | 0,4544 |
| PD vs. PD + PT | 34,52 | -24,49 to 93,54 | No | ns | 0,3481 |
|  |  |  |  |  |  |
| CEJ-ABC analysis | | | | | |
| Tukey's multiple comparisons test | Mean Diff, | 95,00% CI of diff, | Significant? | Summary | Adjusted P Value |
| Control vs. PT | 0,005952 | -0,1001 to 0,1120 | No | ns | 0,9986 |
| Control vs. PD | -0,2255 | -0,3316 to -0,1194 | Yes | **** | <0,0001 |
| Control vs. PD + PT | -0,1124 | -0,2185 to -0,006298 | Yes | * | 0,0351 |
| PT vs. PD | -0,2314 | -0,3375 to -0,1253 | Yes | **** | <0,0001 |
| PT vs. PD + PT | -0,1183 | -0,2244 to -0,01225 | Yes | * | 0,0248 |
| PD vs. PD + PT | 0,1131 | 0,007013 to 0,2192 | Yes | * | 0,0337 |
|  |  |  |  |  |  |
| Tb.Sp analysis | | | | | |
| Tukey's multiple comparisons test | Mean Diff, | 95,00% CI of diff, | Significant? | Summary | Adjusted P Value |
| Control vs. PT | 0,007058 | -0,02135 to 0,03546 | No | ns | 0,9016 |
| Control vs. PD | -0,0308 | -0,05920 to -0,002395 | Yes | * | 0,0301 |
| Control vs. PD + PT | 0,005044 | -0,02336 to 0,03345 | No | ns | 0,9606 |
| PT vs. PD | -0,03786 | -0,06626 to -0,009453 | Yes | ** | 0,0061 |
| PT vs. PD + PT | -0,002014 | -0,03042 to 0,02639 | No | ns | 0,9973 |
| PD vs. PD + PT | 0,03584 | 0,007439 to 0,06425 | Yes | ** | 0,0097 |
|  |  |  |  |  |  |
| Tb.Th analysis | | | | | |
| Tukey's multiple comparisons test | Mean Diff, | 95,00% CI of diff, | Significant? | Summary | Adjusted P Value |
| Control vs. PT | 0,01027 | -0,03338 to 0,05392 | No | ns | 0,9149 |
| Control vs. PD | 0,04454 | 0,0008949 to 0,08819 | Yes | * | 0,0443 |
| Control vs. PD + PT | 0,01181 | -0,03184 to 0,05546 | No | ns | 0,8774 |
| PT vs. PD | 0,03428 | -0,009371 to 0,07793 | No | ns | 0,1615 |
| PT vs. PD + PT | 0,00154 | -0,04211 to 0,04519 | No | ns | 0,9997 |
| PD vs. PD + PT | -0,03274 | -0,07639 to 0,01091 | No | ns | 0,1919 |
|  |  |  |  |  |  |
| BV/TV analysis | | | | | |
| Tukey's multiple comparisons test | Mean Diff, | 95,00% CI of diff, | Significant? | Summary | Adjusted P Value |
| Control vs. PT | 1,003 | -6,951 to 8,957 | No | ns | 0,9852 |
| Control vs. PD | 12,16 | 4,206 to 20,11 | Yes | ** | 0,0016 |
| Control vs. PD + PT | -1,104 | -9,058 to 6,849 | No | ns | 0,9804 |
| PT vs. PD | 11,16 | 3,203 to 19,11 | Yes | ** | 0,0038 |
| PT vs. PD + PT | -2,107 | -10,06 to 5,847 | No | ns | 0,8838 |
| PD vs. PD + PT | -13,26 | -21,22 to -5,311 | Yes | *** | 0,0006 |
